# Role of C-reactive protein in osteoclastogenesis in rheumatoid arthritis

**DOI:** 10.1186/s13075-015-0563-z

**Published:** 2015-03-04

**Authors:** Kyoung-Woon Kim, Bo-Mi Kim, Hee-Won Moon, Sang-Heon Lee, Hae-Rim Kim

**Affiliations:** Conversant Research Consortium in Immunologic disease, Seoul St. Mary’s Hospital, The Rheumatism Research Center, Catholic Research Institute of Medical Science, The Catholic University of Korea, Seoul, South Korea, 505 Banpo-Dong, Seocho-Ku, Seoul, 137-040 Korea; Department of Rheumatology, Research Institute of Medical Science, Konkuk University School of Medicine, 1 Hwayang-dong, Kwangjin-gu, Seoul, 143-729 Korea; Department of Laboratory Medicine, Konkuk University School of Medicine, 1 Hwayang-dong, Kwangjin-gu, Seoul, 143-729 Korea

## Abstract

**Introduction:**

C-reactive protein (CRP) is one of the biomarkers for the diagnosis and assessment of disease activity in rheumatoid arthritis (RA). CRP is not only the by-product of inflammatory response, but also plays proinflammatory and prothrombotic roles. The aim of this study was to determine the role of CRP on bone destruction in RA.

**Methods:**

CRP levels in RA synovial fluid (SF) and serum were measured using the immunoturbidimetric method. The expression of CRP in RA synovium was assessed using immunohistochemical staining. CD14+ monocytes from peripheral blood were cultured with CRP, and receptor activator of nuclear factor-κB ligand (RANKL) expression and osteoclast differentiation were evaluated using real-time PCR, counting tartrate resistant acid phosphatase (TRAP)-positive multinucleated cells and assessing bone resorbing function. CRP-induced osteoclast differentiation was also examined after inhibition of Fcγ receptors.

**Results:**

There was a significant correlation between CRP levels in serum and SF in RA patients. The SF CRP level was correlated with interleukin (IL)-6 levels, but not with RANKL levels. Immunohistochemical staining revealed that compared with the osteoarthritis synovium, CRP was more abundantly expressed in the lining and sublining areas of the RA synovium. CRP stimulated RANKL production in monocytes and it induced osteoclast differentiation from monocytes and bone resorption in the absence of RANKL.

**Conclusions:**

CRP could play an important role in the bony destructive process in RA through the induction of RANKL expression and direct differentiation of osteoclast precursors into mature osteoclasts. In the treatment of RA, lowering CRP levels is a significant parameter not only for improving disease activity but also for preventing bone destruction.

## Introduction

Rheumatoid arthritis (RA) is a systemic inflammatory disease characterized by synovitis of peripheral joints and subsequent joint destruction. Assessment of disease activity is based on the count of tender and swollen joints, the measurement of erythrocyte sedimentation rate (ESR) or serum levels of acute phase reactants such as C-reactive protein (CRP), and the pain score of patients [1-3]. The new classification criteria for RA including ESR and CRP measurements allow early aggressive treatment of RA [4,5]. As an inflammatory biomarker for RA, CRP correlates with disease activity, histological changes in the synovium, and radiological progression, responding very quickly to changes in disease activity [6-10]. In terms of clinical parameters, CRP level correlates with morning stiffness, pain, fatigue, grip strength, articular index, and disability [7]. Moreover, CRP level is not affected by age, gender, and abnormalities in erythrocytes and serum proteins. Hence, among various parameters, CRP is the most reliable and objective measure and a useful prognostic factor for disease progression to joint damage and functional outcome [11-13].

CRP is a member of the pentraxin protein family, which is composed of five 23-kDa subunits and it can increase by 1,000-fold or more with infection, inflammation, and tissue injury [14,15]. Interleukin (IL)-6, IL-1β, and tumor necrosis factor (TNF)-α promote the synthesis of CRP in hepatocytes [15,16]. While hepatocytes are main sources of CRP, monocytes, lymphocytes, adipocytes, neurons, and vascular smooth muscle cells are the extrahepatic sources of CRP [17-19]. To eliminate infectious microorganisms and damaged or dead cells, CRP activates the classical complement cascade and stimulates the influx and phagocytic activity of neutrophils [20,21]. In addition to clearing infection, CRP plays a regulatory role in inflammation and atherosclerotic thrombosis. It stimulates endothelial cells to produce intercellular adhesion molecule (ICAM)-1, vascular cell adhesion molecule (VCAM)-1, E-selectin, and monocyte chemotactic protein (MCP)-1 and inhibits the expression of endothelial nitric oxide synthase [22-24]. Interaction of CRP with Fcgamma receptor (FcγR)I and FcγRIIA also promotes the production of proinflammatory cytokines, resulting in the amplification loop of inflammatory reaction [25]. Until now, CRP has been in the spotlight as a marker of disease activity and a by-product of inflammatory reactions in RA. However, it is possible that CRP is one of the proinflammatory and bone destruction molecules in the pathogenesis of RA.

In this study, we hypothesized that CRP not only results from the inflammation process, but also triggers proinflammatory responses and bone destruction. These processes are initiated through the induction of the receptor activator of nuclear factor-κB ligand (RANKL) protein and direct stimulation of osteoclastogenesis, causing a vicious loop between inflammation and bone destruction in RA. We studied the effects of CRP on the induction of RANKL and osteoclast differentiation from peripheral blood monocytes. We found that CRP induced RANKL expression in peripheral blood monocytes and osteoclast precursors and stimulated these cells to differentiate into osteoclasts. In the treatment of RA, the reduction of CRP level reflects not only the control of disease activity, but also the prevention of bone destruction.

## Materials and methods

### Patients

Synovial tissues were obtained from seven patients with RA (mean age 63.4 ± 4.6 years) and five patients with osteoarthritis (OA) (mean age 56.6 ± 4.7 years), who were undergoing total knee replacement surgery. Synovial fluid and serum were obtained from 20 RA patients who fulfilled the 1987 revised criteria of the American College of Rheumatology [26]. Informed consent was obtained from all patients, and the experimental protocol was approved by the Konkuk University Medical Center Human Research Ethics Committee.

### Reagents

Recombinant CRP was supplied by Sigma-Aldrich, St Louis, MO, USA. Recombinant macrophage colony-stimulating factor (M-CSF) was purchased from R&D Systems (Minneapolis, MN, USA). Anti-CD64, anti-CD32 and anti-CD16 were obtained from R&D Systems. Human immunoglobulin G (IgG) was purchased from Green Cross Corp. (Yong-In, Korea).

### Measurement of CRP

High-sensitivity CRP was measured by an immunoturbidimetric method (CRP II Latex X2, Denka Seiken Co. Ltd., Tokyo, Japan) using an autoanalyzer (Toshiba, Tokyo, Japan). Measurement range of this assay is 0.01 to 32 mg/dL.

### Enzyme-linked immunosorbent assay of RANKL, IL-6, IL-1β and TNF-α

Briefly, a 96-well plate (Nunc) was coated overnight with 4 μg/ml monoclonal antibodies against RANKL, IL-6, IL-1β and TNF-α (R&D Systems) at 4°C. After blocking with phosphate-buffered saline (PBS)/1% bovine serum albumin (BSA)/0.05% Tween 20 for 2 hours at room temperature (22 to 25°C), the test samples and standard recombinant RANKL, IL-6, IL-1β and TNF-α were added to the 96-well plate and incubated at room temperature for 2 hours. The plates were washed four times with PBS/Tween 20, and then incubated with 500 ng/ml biotinylated mouse monoclonal antibodies against RANKL, IL-6, IL-1β and TNF-α for 2 hours at room temperature. After washing, streptavidin-alkaline phosphate-horseradish peroxidase conjugate (Sigma-Aldrich) was added and the plate was incubated for 2 hours, after which the plate was washed again and incubated with 1 mg/ml *p*-nitrophenyl phosphate (Sigma-Aldrich) dissolved in diethanolamine (Sigma-Aldrich) to develop the color reaction. The reaction was quenched by the addition of 1 M NaOH, and the optical density of each well was read at 405 nm. The lower detection limit of RANKL, IL-6, IL-1β and TNF-α was 10 pg/ml. Recombinant human RANKL, IL-6, IL-1β and TNF-α were diluted in the culture medium and used as the calibration standard with concentration of 10 to 2,000 pg/ml. A standard curve was drawn by plotting the optical density as a function of the log of the concentration of recombinant cytokines, and was used to calculate the RANKL, IL-6, IL-1β and TNF-α concentrations in the test samples.

### Immunohistochemical analysis of RA synovial tissues

Immunohistochemical staining for CRP was performed on sections of synovial tissues. Briefly, synovial tissues were obtained from patients with RA and OA, fixed with 4% paraformaldehyde solution overnight at 4°C, dehydrated with a graded series of alcohol, washed, embedded in paraffin, and cut into 7-μm thick sections. The sections were depleted of endogenous peroxidase activity by adding methanolic H_2_O_2_ and blocked with normal serum for 30 minutes. After overnight incubation at 4°C with polyclonal anti-human CRP antibody (Santa Cruz Biotechnology, Santa Cruz, CA, USA), the samples were incubated with the secondary antibody, biotinylated anti-rabbit IgG, for 20 minutes and incubated with the streptavidin-peroxidase complex (Vector Laboratories Ltd., Peterborough, UK) for 1 hour followed by incubation with 3,3′-diaminobenzidine (Dako, Glostrup, Denmark) for 5 minutes. The sections were counterstained with hematoxylin. The samples were photographed using an Olympus photomicroscope (Tokyo, Japan). For the evaluation of immunohistochemistry slides using ImageJ (NIH, Bethesda, MD, USA), images are captured onto the hard drive of the workstation computer. Thereafter, captured images are opened in NIH Image/ImageJ for evaluating indices of positivity on immunohistochemistry slides.

### Monocyte isolation

Peripheral blood mononuclear cells (PBMC) were separated by Ficoll-Hypaque (Sigma-Aldrich) density gradient centrifugation from the buffy coats obtained from healthy volunteers. The cells were washed three times with sterile PBS and resuspended in RPMI 1640 (Life Technologies, Grand Island, NY, USA) supplemented with 10% fetal bovine serum (FBS), 2 mM L-glutamine, and 1% penicillin-streptomycin, henceforth called complete medium. Freshly isolated PBMC were incubated at 37°C in complete medium and allowed to adhere for 45 minutes. The nonadherent cells were removed and the adherent cells were washed with sterile PBS, harvested with a rubber policeman, and stained with the monocyte-specific anti-CD14 monoclonal antibody to assess the purity of the preparation. Ninety percent of the isolated cells expressed CD14. The osteoclast precursors were prepared using the monocytes-enriched fraction from the peripheral blood.

### Expression of target mRNA determined by real-time PCR with SYBR Green I

Monocytes were stimulated with various CRP concentrations (0.1, 0.5, or 1 ug/ml). For signal pathway analysis of RANKL, the monocytes were incubated in the presence or absence of anti-CD64, anti-CD32 and anti-CD16 (10 ng/ml) for 1 hour before the addition of recombinant human (rh) CRP (rhCRP). After incubation for 12 hours, mRNA was extracted using RNAzol B (Biotex Laboratories, Houston, TX, USA) according to the manufacturer’s instructions. The reverse transcription of 2 μg of total mRNA was performed at 42°C using the Superscript™ reverse transcription system (Takara Bio Inc., Shiga, Japan). Real-time PCRs were performed in 20-μl final volumes in capillary tubes in a LightCycler instrument (Roche Diagnostics, Mannheim, Germany). The following primers were used for each molecule: for RANKL, 5′-ACC-AGC-ATC-AAA-ATC-CCA-AG-3′ (sense) and 5′-CCC-CAA-AGT-ATG-TTG-CAT-CC-3′(antisense); for FcγR1a, 5′-TGA-GGT-GTC-ATG-CGT-GGA-A-3′ (sense) and 5′-GGT-AGG-TGC-CAT-TGT-GAC-TTA-TG-3′ (antisense); for FcγR2a, 5′-ATC-ATT-GTG-GCT-GTG-GTC-ATT-G-3′ (sense) and 5′-CCA-ACA-ATG-ACT-ATG-AAA-CAG-CTG-AC-3′ (antisense); for FcγR2b, 5′-CCT-GAT-GAC-CAG-AAC-CGT-ATT-TAG-T-3′ (sense) and 5′-TTT-TGG-TTC-TGC-AGC-ATC-TCC-3′ (antisense); for CTR, 5′-TGG-TGC-CAA-CCA-CTA-TCC-ATG-C-3′ (sense) and 5′-CAC-AAG-TGC-CGC-CAT-GAC-AG-3′ (antisense); for cathepsin K, 5′-TGA-GGC-TTC-TCT-TGG-TGT-CCA-TAC-3′ (sense) and 5′-AAA-GGG-TGT-CAT-TAC-TGC-GGG-3′ (antisense); for MMP-9, 5′-CGC-AGA-CAT-CGT-CAT-CCA-GT-3′ (sense) and 5′- GGA-TTG-GCC-TTG-GAA-GAT-GA-3′ (antisense); for RANK, 5′-GCT-CTA-ACA-AAT-GTG-AAC-CAG-GA-3′ (sense) and 5′-GCC-TTG-CCT-GTA-TCA-CAA-ACT-3′ (antisense); for TRAP, 5′-GAC-CAC-CTT-GGC-AAT-GTC-TCT-G-3′ (sense) and 5′-TGG-CTG-AGG-AAG-TCT-CTG-AGT-TG-3′ (antisense); for DC-STAMP, 5′-TTC-GCT-CGT-CCT-GCT-TGG-3′ (sense) and 5′-GCG-GGA-TGT-CTG-GTG-ATG-TAG-3′ (antisense); for MFR, 5′-CCC-AGG-GCT-CCA-CTT-CTT-C-3′ (sense) and 5′-TGT-GGT-TGT-TGG-GCT-CCG-3′ (antisense); for β-actin, 5′-GGA-CTT-CGA-GCA-AGA-GATGG- 3′ (sense) and 5′-TGT-GTT-GGC-GAT-CAG-GTCTTT- G-3′ (antisense).

Reaction mixtures contained 2 μl of LightCycler FastStart DNA mastermix for SYBR Green I (Roche Diagnostics), 0.5 μM each primer, 4 mM MgCl2, and 2 μl of template DNA.

All capillaries were sealed, centrifuged at 500 *g* for 5 seconds, and then amplified in a LightCycler instrument, with activation of polymerase (95°C for 10 minutes), followed by 45 cycles of 10 seconds at 95°C, 10 seconds at 60°C, and 10 seconds at 72°C.

The temperature transition rate was 20°C/second for all steps. Double-stranded PCR product was measured during the 72°C extension step by detection of fluorescence associated with the binding of SYBR Green I to the product. Fluorescence curves were analyzed with LightCycler software, version 3.0. For quantification analysis of target mRNA, LightCycler was used. Relative expression levels of samples were calculated by normalizing target levels to the endogenously expressed housekeeping gene (beta-actin). Melting curve protocol under the following conditions: 0 second (hold time on reaching temperatures) at 95°C, 15 seconds at 65°C, and 0 second (hold time) at 95°C. The temperature change rate was 20°C/second, except in the final step, in which it was 0.1°C/second. The melt peak generated represented the specific amplified product. The crossing point was defined as the maximum of the second derivative from the fluorescence curve. Negative controls, which contained all the elements of the reaction mixture except template DNA, were also included. All samples were processed in duplicate.

### Western blot analysis

Monocytes were incubated with CRP. After incubation for 72 hours, whole-cell lysates were prepared from about 5 × 10^6^ cells by homogenization in the lysis buffer, and centrifuged at 14,000 rpm for 15 minutes. The protein concentration in the supernatant was determined using the Bradford method (Bio-Rad, Hercules, CA, USA). Protein samples were separated on 10% sodium dodecyl sulfate-polyacrylamide electrophoresis (SDS-PAGE), and transferred to a nitrocellulose membrane (Amersham Pharmacia Biotech, Uppsala, Sweden). For western hybridization, the membrane was preincubated with 0.5% skim milk in 0.1% Tween 20 in Tris-buffered saline (TTBS) at room temperature for 2 hours. The primary antibody to p-syk, p-Akt, Akt, p-ERK, ERK, p-JNK, JNK, p-p38, p38, beta-actin (Cell Signaling Beverly, MA, USA), diluted 1:1000 in 5% BSA-0.1% Tween 20/TBS, was added and incubated for overnight at 4°C. The membrane was washed four times with TTBS, and horseradish peroxidase-conjugated secondary antibody was added and incubated for 1 hour at room temperature. After TTBS washing, hybridized bands were detected using the ECL detection kit and Hyperfilm-ECL reagents (Amersham Pharmacia).

### Osteoclast differentiation

PBMC were isolated by Ficoll-Hypaque (Sigma-Aldrich, Poole, Dorset, UK) density gradient centrifugation from the buffy coats obtained from healthy volunteers. The cells were washed three times with sterile PBS and resuspended in RPMI 1640 (Life Technologies) supplemented with 10% FBS, 2 mM L-glutamine, and 1% penicillin-streptomycin (complete medium). Freshly isolated PBMC were incubated at 37°C in complete medium and allowed to adhere for 45 minutes. The nonadherent cells were removed and the adherent cells were washed with sterile PBS, harvested with a rubber policeman, and stained with monocyte-specific anti-CD14 monoclonal antibody to assess the purity of the preparation. Ninety percent of the isolated cells expressed CD14. The osteoclast precursors were prepared using the monocyte-enriched fraction from the peripheral blood. The cells were cocultured for three weeks in minimal essential medium (MEM)-α and 10% heat-inactivated FBS in the presence of 25 ng/ml rhM-CSF. The medium was changed on day 3 and then every other day thereafter. On day 21, tartrate-resistant acid phosphatase (TRAP)-positive cells were identified using a leukocyte acid phosphatase kit according to the manufacturer’s protocol (Sigma-Aldrich).

### Bone resorbing function assay

We performed an *in vitro* resorption pit assay using a bone resorption assay kit (Cosmo Bio Co., Ltd., Tokyo, Japan). Monocytes were cultured on a bone-coated plate with M-CSF in the presence or absence of various concentration of CRP for 14 days. The cells were removed from a bone-coated plate by wiping the surface of it. The number of pits formed by bone resorption on the plate was counted.

### Statistical analysis

Results are expressed as mean ± standard error of the mean (SEM). Statistical differences were assessed using one-way or two-way repeated-measures analysis of variance (ANOVA), followed by the Dunnett’s multiple comparison test or Student’s *t* test for comparison of each group. A *P* value <0.05 was considered significant.

## Results

### CRP levels in RA serum and SF and their correlation with RANKL

**Figure 1:**
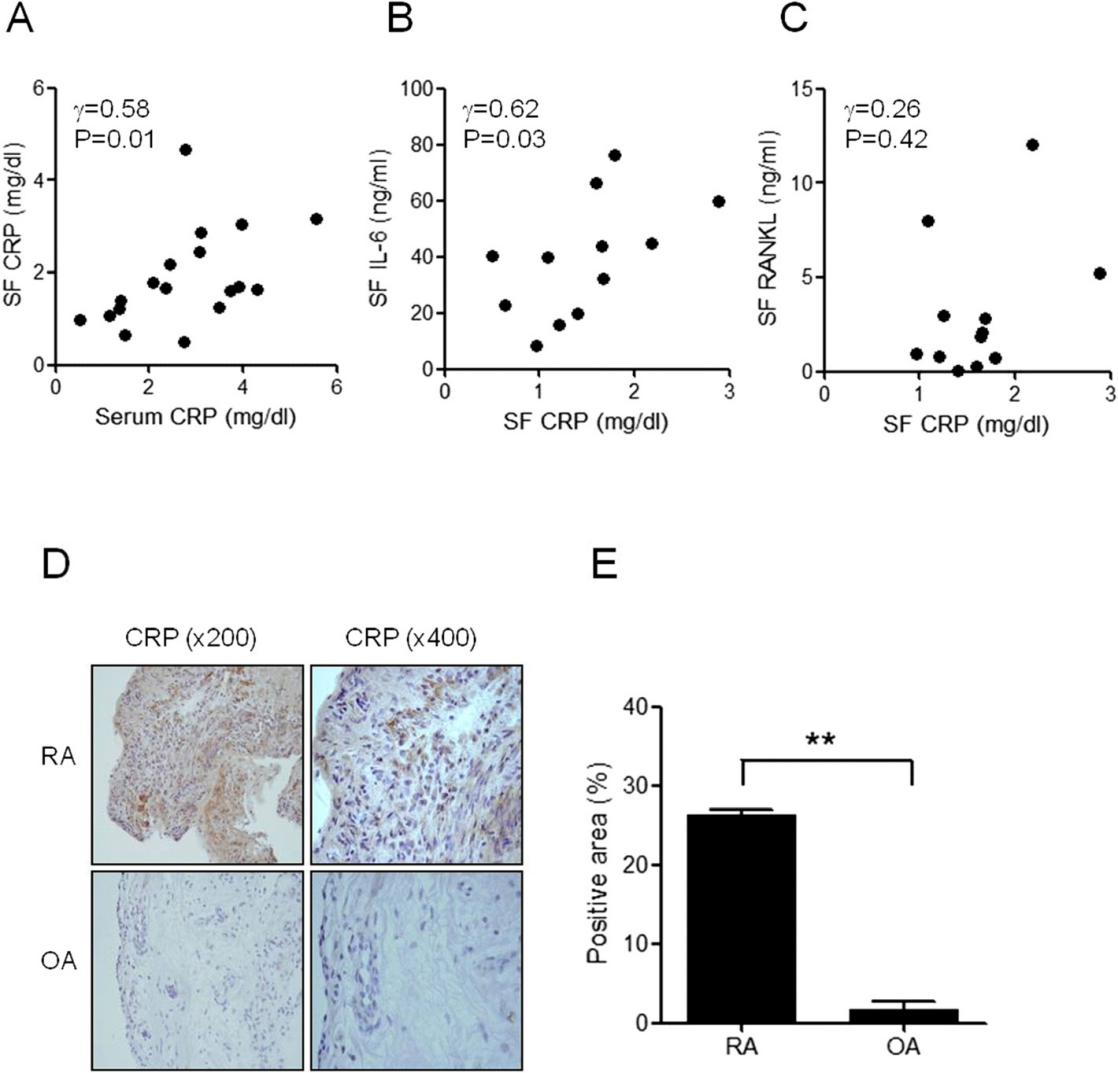
**The expression of CRP in serum, synovial fluid (SF), and synovial tissues of RA patients. (A)** SF and serum of 20 RA patients were collected and CRP level was measured using an immunoturbidimetric method. There is significant correlation between SF and serum levels of CRP (R^2^ =  0.58, *P* <0.05). **(B)** SF IL-6 and IL-6 levels were measured using sandwich ELISA. SF CRP level is correlated with SF IL-6 level (R^2^ = 0.62, *P* <0.05). **(C)** There is no correlation between SF CRP and SF RANKL levels in RA patients. **(D)** Immunohistochemical staining for CRP in the synovium of patients with RA and osteoarthritis (OA) shows abundant expression of CRP in the synovial lining and sublining areas in RA synovium, while there is minimal expression of CRP in OA synovium (original magnification: 200× and 400×). The figures are representatives of three independent experiments. **(E)** The quantitative measurement of positive area using ImageJ is larger in RA synovium than OA synovium (original magnification; 400×). ^**^ *P* <0.01. CRP, C-reactive protein; ELISA, enzyme-linked immunosorbent assay; IL, interleukin; RA, rheumatoid arthritis; RANKL, receptor activator of nuclear factor kappa-B ligand.

Serum and synovial fluid (SF) CRP levels of 20 RA patients were measured using an immunoturbidimetric method. The clinical characteristics of the RA patients are as follows: 17 females and 3 males, age 57.5 ± 3.7 years (range: 26 to 87 years), disease duration 3.5 ± 0.7 years (range: 0.1 to 12 years), ESR 51.1 ± 7.1 mm/h (range: 8 to 116 mm/h, normal <15), serum CRP 2.7 ± 0.3 mg/dL (range: 0.5 to 5.6 mg/dL, normal <0.3), SF CRP 1.9 ± 0.2 mg/dL (range: 0.5 to 4.7 mg/dL), rheumatoid factor 70.9 ± 16.9 IU/mL (range: 3 to 266 IU/mL, normal <18) and anti-cyclic citrullinated protein antibody 64.1 ± 23.6 U/mL (range: 0.3 to 338 U/mL, normal <5). In 20 OA patients, the serum CRP level was 0.08 ± 0.02 mg/dL (range: 0.01 to 0.3 mg/dL) and the SF CRP level was 0.04 ± 0.01 mg/dL (range: 0.02 to 0.09 mg/dL); these values were significantly lower than those in RA patients (*P* <0.005, data not shown). There was no difference of SF CRP levels between the patients who were taking steroids and the patients who did not (9.23 mg/dl vs. 11. 67 mg/dl, *P* = 0.38). There was significant positive correlation between serum and SF CRP levels in RA patients (R^2^ = 0.58, *P* <0.05, Figure 1A). Both serum and SF CRP levels were correlated with the SF IL-6 level (R^2^ = 0.78, *P* <0.005 and R^2^ = 0.62, *P* <0.05, respectively); however, there was no correlation between SF CRP and SF RANKL levels (Figure 1B, C). Immunohistochemical staining revealed that compared with the OA synovium, CRP was more abundantly expressed in the lining and sublining areas of the RA synovium (Figure 1D, E).

### The effect of CRP on the expression of RANKL mRNA in osteoclast precursors

**Figure 2:**
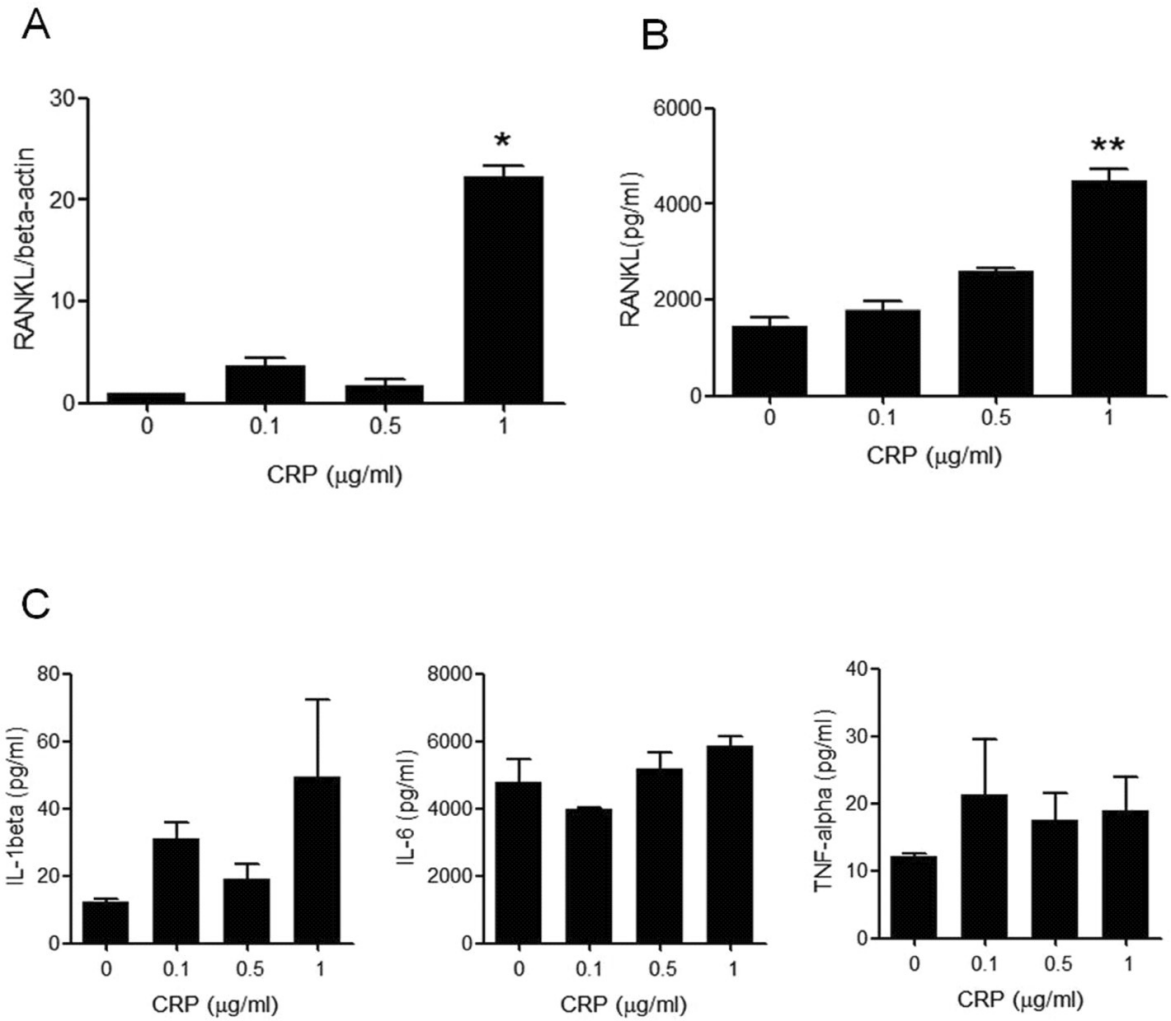
**CRP-induced RANKL expression and production in peripheral blood monocytes. (A)** After peripheral blood CD14+ monocytes were cultured with 0 to 1.0 μg/mL of CRP for 72 h, the expression of RANKL mRNA was determined using real-time PCR. The expression of RANKL mRNA increased in a dose-dependent manner with a maximal effect at 1.0 μg/mL of CRP. **(B)** In monocytes cultured with CRP, the production of RANKL was measured using sandwich ELISA. CRP also increased RANKL production of cultured monocytes in a dose-dependent manner. **(C)** After peripheral blood CD14+ monocytes were cultured with CRP for 72 h, the concentrations of IL-1β, TNF-α, and IL-6 in culture media were determined using sandwich ELISA. CRP does not stimulate monocytes to produce IL-1β, IL-6, or TNF-α. The data represents the mean ± SEM for three independent experiments; ^*^ *P* <0.05, ^**^ *P* <0.01. CRP, C-reactive protein; ELISA, enzyme-linked immunosorbent assay; IL, interleukin; RANKL, receptor activator of nuclear factor kappa-B ligand; SEM, standard error of the mean; TNF, tumor necrosis factor.

After peripheral blood CD14+ monocytes were stimulated with various doses of CRP, RANKL mRNA expression and protein production were determined using real-time PCR and enzyme-linked immunosorbent assay (ELISA) in the culture medium, respectively. CRP increased the expression of RANKL mRNA in a dose-dependent manner with maximal effect at a concentration of 1 μg/mL (Figure 2A). In the culture medium, the production of RANKL also increased with CRP stimulation, following a pattern similar to that of the gene expression (Figure 2B). However, CRP did not induce the production of proinflammatory cytokines such as IL-1β, TNF-α, or IL-6 (Figure 2C).

### Mediation of Fcgamma receptors (FcγRs) I and II in the CRP-induced RANKL expression

**Figure 3:**
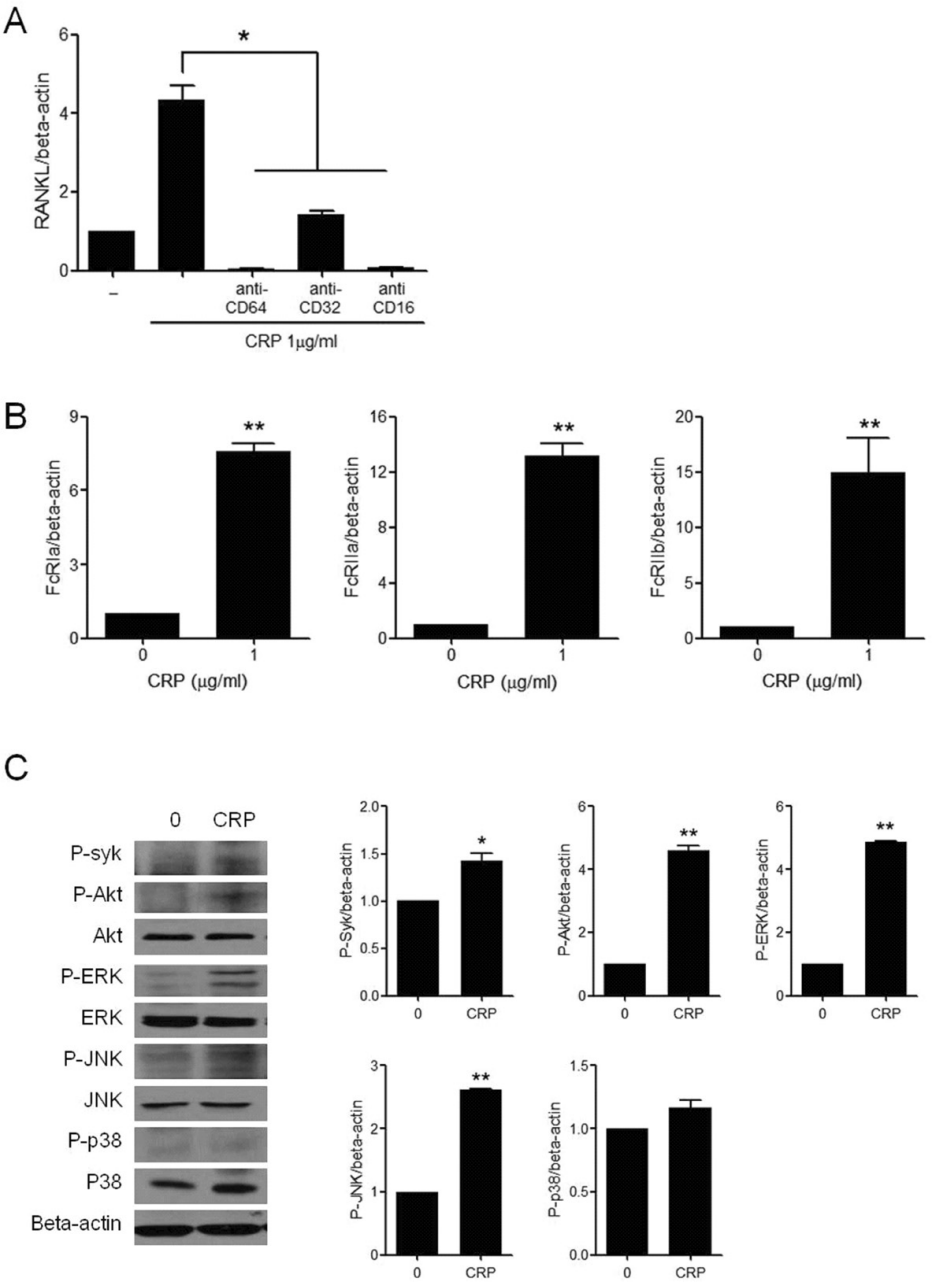
**The effect of FcγR on CRP-induced RANKL expression in monocytes. (A)** Peripheral blood CD14+ monocytes were pretreated with anti-CD64 (FcγRI inhibitor), anti-CD32 (FcγRIIa inhibitor), or anti-CD16 (FcγRIIb inhibitor) for 1 h and were cultured with 1 μg/mL of CRP for 72 h. The expression of RANKL mRNA was determined using real-time PCR. The expression of RANKL mRNA decreased after inhibition of FcγRI, FcγRIIa, and FcγRIIb. **(B)** After peripheral blood CD14+ monocytes were cultured with 1 μg/mL of CRP for 72 h, the gene expressions of FcγRI, FcγRIIa, and FcγRIIb were determined using real-time PCR. The expressions of FcγRI, FcγRIIa and FcγRIIb mRNA were increased by CRP stimulation. **(C)** CRP-induced expression of intracellular signal molecules was determined by Western blotting. The amount of protein expression was normalized to beta-actin, and the ratio of the phosphorylated form to the total form was calculated. CRP increased the phosphorylation of Syk, Akt, ERK and JNK. The data represents the mean ± SEM for three independent experiments; ^*^ *P* <0.05 and ^**^ *P* <0.01. CRP, C-reactive protein; FcγR, Fcgamma receptors; RANKL, receptor activator of nuclear factor kappa-B ligand; SEM, standard error of the mean; TNF, tumor necrosis factor.

To define the predominant receptor involved in the CRP-induced RANKL expression, peripheral blood monocytes were cultured with CRP in the presence of FcγR inhibitors. After the monocytes were cultured with anti-FcγRI (CD64), anti-FcγRIIA (CD32), or anti-FcγRIIB (CD16) in the presence of CRP, the gene expression of RANKL decreased with all three receptor inhibitors (Figure 3A). The expression of FcγRI, FcγRIIa and FcγRIIb was significantly increased by CRP stimulation (Figure 3B). To define downstream pathways of the FcγR, the phosphorylation of Syk, Akt, ERK, JNK and p38 was determined by Western blot analysis and CRP increased the phosphorylation of Syk, Akt, ERK and JNK (Figure 3C).

### The effect of CRP on osteoclast differentiation

**Figure 4:**
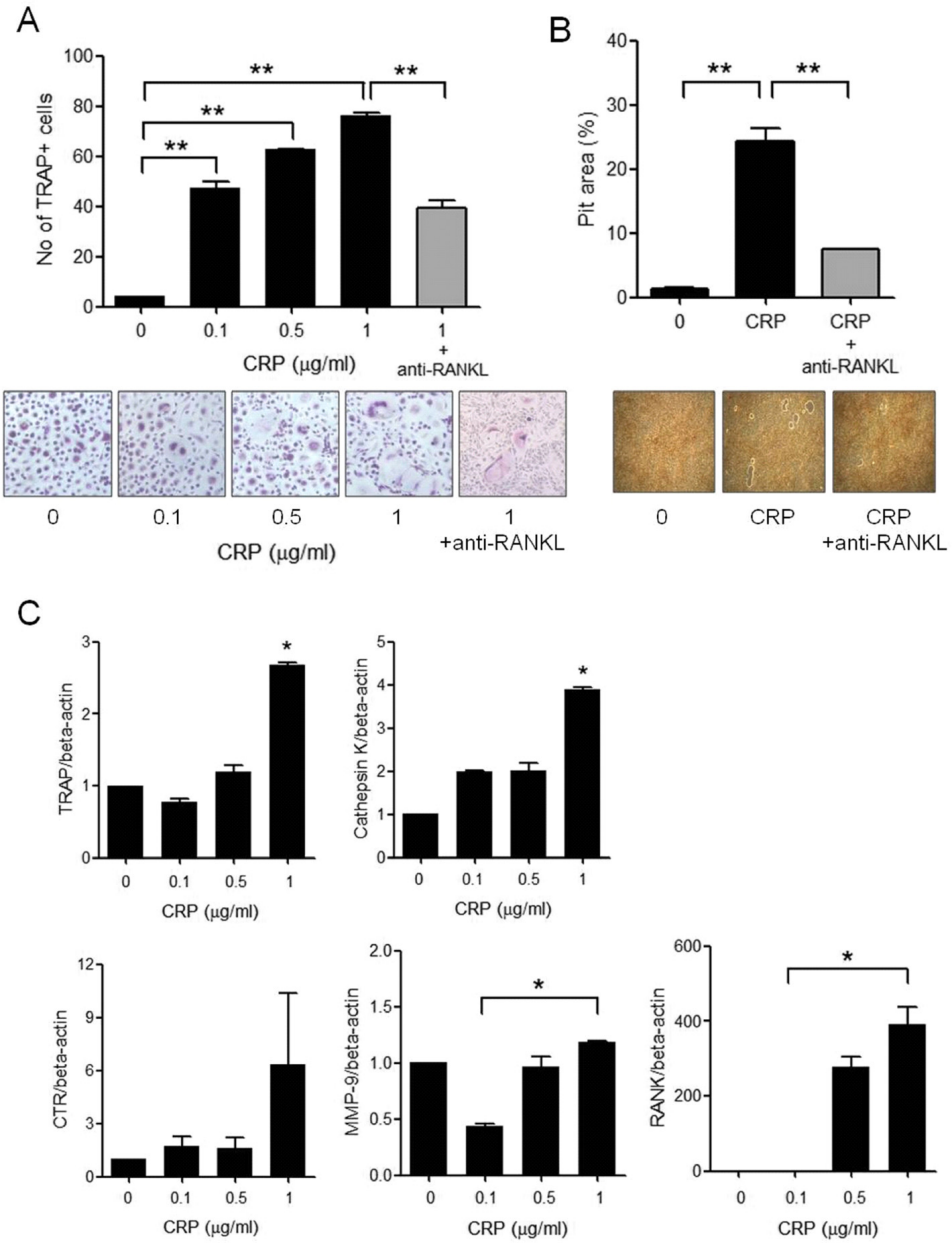
**CRP-induced osteoclast differentiation from CD14+ monocytes isolated from peripheral blood. (A)** CD14+ monocytes, which were isolated from peripheral blood, were cultured with 25 ng/mL of M-CSF and 0 to 1.0 μg/mL of CRP in the presence or absence of anti-RANKL. After 21 days of culture, TRAP-positive multinucleated cells were counted. TRAP+ multinucleated osteoclasts were differentiated from the monocytes in a dose-dependent manner with a maximal effect at 1.0 μg/mL of CRP and anti-RANKL partially reduced the CRP-induced osteoclastogenesis. The figures represent one of three independent experiments (original magnification: 200×). **(B)** Monocytes were cultured on a bone-coating plate with M-CSF, CRP and anti-RANKL for 21 days. The number of pits formed by bone resorption on the plate was counted. CRP significantly increased the bone resorbing function and this CRP-induced bone resorption was incompletely inhibited by neuralization of RANKL. The figures represent one of three independent experiments (original magnification: 200×). **(C)** The gene expressions of osteoclast markers such as TRAP, cathepsin K, CTR, MMP-9, and RANK were measured from differentiated osteoclasts using real-time PCR. The gene expressions of TRAP, cathepsin K, MMP-9, and RANK increased significantly with CRP stimulation. The data represents the mean ± SEM for three independent experiments; ^*^ *P* <0.05 and ^**^ *P* <0.01. CRP, C-reactive protein; M-CSF, macrophage colony-stimulating factor; MMP, matrix metalloproteinases; RANKL, receptor activator of nuclear factor kappa-B ligand; SEM, standard error of the mean; SF, synovial fluid; TRAP, tartrate-resistant acid phosphatase.

Peripheral blood monocytes can differentiate into TRAP+ multinucleated osteoclasts in the presence of RANKL and M-CSF [27]. To determine the direct and independent effect of CRP on the induction of osteoclastogenesis, peripheral blood CD14+ monocytes were isolated and cultured with CRP and M-CSF in the absence of RANKL. After 21 days of culture, TRAP+ multinucleated osteoclasts were differentiated from the monocytes in a dose-dependent manners and functional bone resorption was induced in the CRP and M-CSF culture systems, which did not contain RANKL. CRP stimulated osteoclast differentiation in a dose-dependent manner (Figure 4A) and the CRP-induced osteoclastogenesis and bone resorbing function were reduced incompletely by inhibition of RANKL (Figure 4A, B). The gene expressions of TRAP, cathepsin K, matrix metalloproteinase (MMP)-9, and RANK also increased in the differentiated osteoclasts (Figure 4C).

### Mediation of FcγRs in the CRP-induced osteoclastogenesis

**Figure 5:**
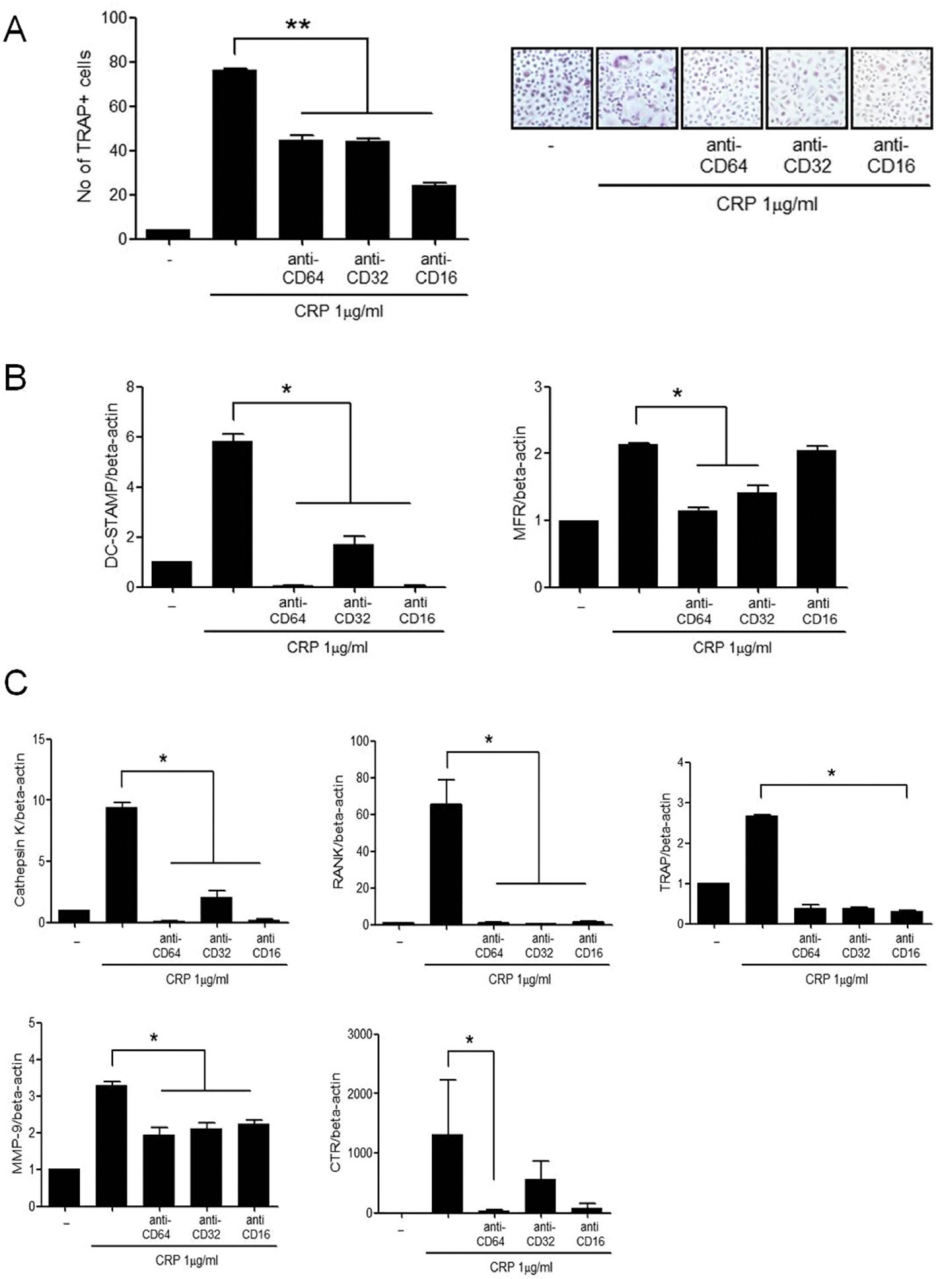
**The effect of FcγRs on the CRP-induced osteoclastogenesis from monocytes. (A)** CD14+ monocytes, which were isolated from peripheral blood, were cultured with 25 ng/mL of M-CSF and 1.0 μg/mL of CRP in the presence of anti-CD64 (FcγRI inhibitor), anti-CD32 (FcγRIIa inhibitor), or anti-CD16 (FcγRIIb inhibitor). After 21 days of culture, TRAP+ multinucleated cells were counted. The inhibition of FcγRI, FcγRIIa, and FcγRIIb decreased CRP-induced osteoclastogenesis. The figure represents one of three independent experiments (original magnification: 200×). **(B)** The gene expression of osteoclast fusion proteins such as DC-STAMP and MFR were measured from differentiated osteoclasts using real-time PCR. The expression of DC-STAMP decreased significantly with the inhibition of FcγRI, FcγRIIa, and FcγRIIb, and the expression of MFR decreased significantly with the inhibition of FcγRI and FcγRIIa. **(C)** The gene expressions of osteoclast markers such as cathepsin K, RANK, MMP-9, and CTR were measured from differentiated osteoclasts using real-time PCR. The gene expressions of cathepsin K, RANKL, and MMP-9 decreased significantly with the inhibition of FcγRI, FcγRIIa, and FcγRIIb. The expression of CTR mRNA decreased with the inhibition of FcγRI. The data represents the mean ± SEM for three independent experiments; ^*^ *P* <0.05 and ^**^ *P* <0.01. CRP, C-reactive protein; CTR, calcitonin receptor; DC-STAMP, dendritic cell-specific transmembrane protein; FcγR, Fcgamma receptors; M-CSF, macrophage colony-stimulating factor; MFR, macrophage fusion receptor; MMP, matrix metalloproteinases; RANKL, receptor activator of nuclear factor kappa-B ligand; SEM, standard error of the mean.

After the monocytes were cultured with anti-CD64, anti-CD32, or anti-CD16 in the presence of CRP and M-CSF, the CRP-induced osteoclast differentiation were partially decreased (Figure 5A). The gene expressions of osteoclast fusion proteins and dendritic cell-specific transmembrane protein (DC-STAMP) decreased with all inhibitors, and the gene expression of MFR decreased with the inhibitors of FcγRI and FcγRIIa (Figure 5B). The gene expression of RANK, MMP and cathepsin K also decreased with all three inhibitors and the gene expression of calcitonin receptor (CTR) decreased with the FcγRI inhibitor (Figure 5C).

### The effect of a representative FcγR ligand, IgG, on RANKL expression and osteoclast differentiation

**Figure 6:**
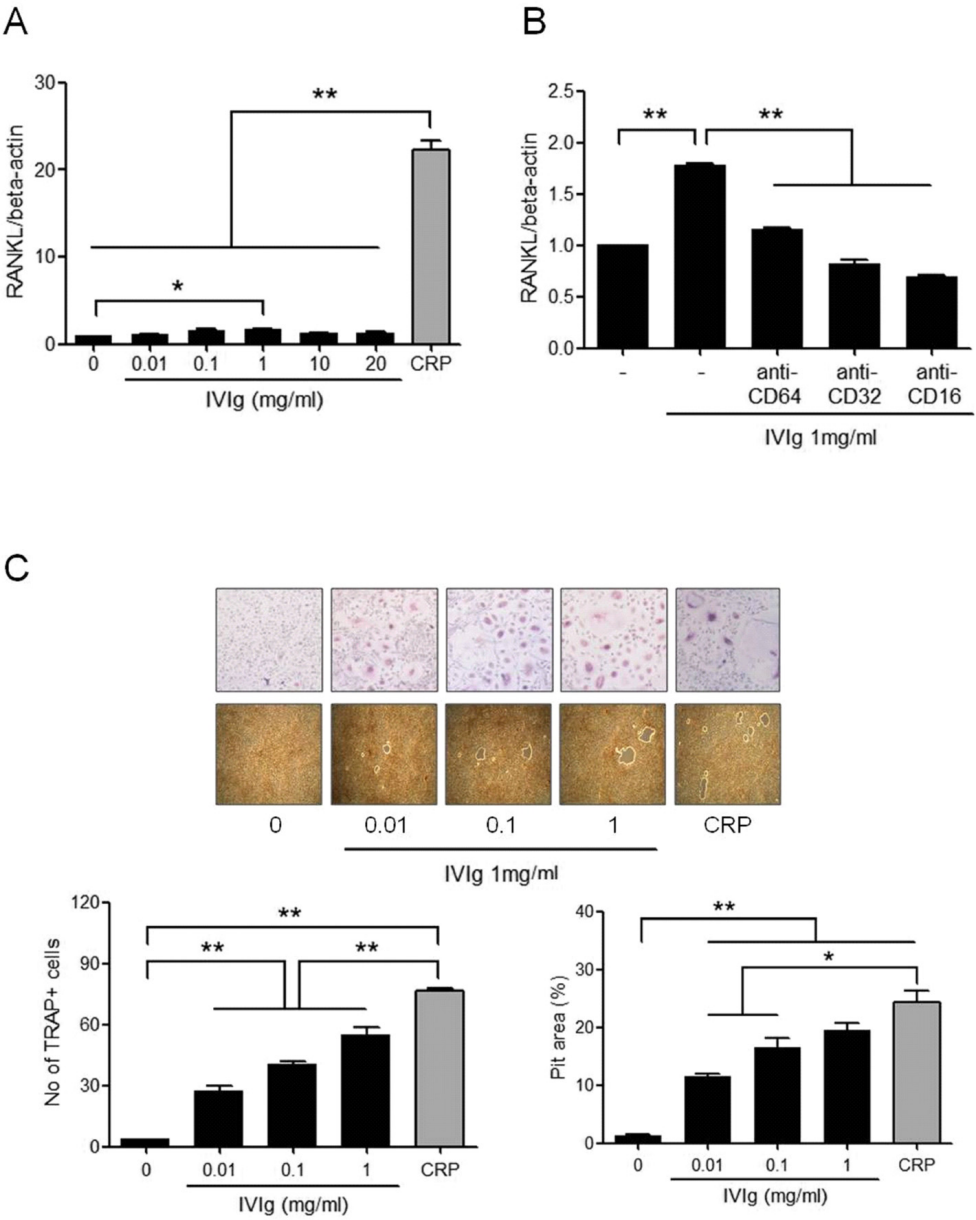
**Immunoglobulin G (IgG)-induced RANKL expression in peripheral blood monocytes and osteoclast differentiation. (A)** After peripheral blood CD14+ monocytes were cultured with 0 to 20 mg/mL of intravenous immunoglobulin (IVIg) for 72 h, the expression of RANKL mRNA was determined using real-time PCR. The expression of RANKL mRNA increased at 1.0 mg/mL of IVIg. **(B)** Peripheral blood CD14+ monocytes were pretreated with anti-CD64, anti-CD32, or anti-CD16 for 1 h and were cultured with 1 mg/mL of IVIg for 72 h. The expression of RANKL mRNA was determined using real-time PCR. The expression of RANKL mRNA decreased after inhibition of FcγRI, FcγRIIa, and FcγRIIb. **(C)** CD14+ monocytes, which were isolated from peripheral blood, were cultured with 25 ng/mL of M-CSF and 0 to 1.0 mg/mL of IVIg. After 21 days of culture, TRAP-positive multinucleated cells were counted and bone resorbing function was assessed. TRAP+ multinucleated osteoclasts were differentiated from the monocytes and bone resorbing function was induced by IVIg, however, the effect was smaller than that of CRP. The figures represent one of three independent experiments (original magnification: 200×). The data represents the mean ± SEM for three independent experiments; ^*^ *P* <0.05, ^**^ *P* <0.01. CRP, C-reactive protein; FcγR, Fcgamma receptors; M-CSF, macrophage colony-stimulating factor; RANKL, receptor activator of nuclear factor kappa-B ligand; SEM, standard error of the mean; TRAP, tartrate-resistant acid phosphatase.

FcγRs are receptors for the Fc portion of immunoglobulins, we compared the effect of CRP with IgG on RANKL expression and osteoclastogenesis. Human IgG also stimulated peripheral blood monocytes to express RANKL mRNA, but CRP showed larger impact on the RANKL expression than IgG (Figure 6A). After the monocytes were cultured with anti-CD64, anti-CD32, or anti-CD16 in the presence of IgG, the gene expression of RANKL decreased with all three receptor inhibitors (Figure 6B). IgG, in the presence of M-CSF, also induced TRAP+ multinucleated osteoclasts from the monocytes and bone resorption, however, the number of TRAP+ osteoclasts from the culture of IgG was smaller than that from the culture of CRP (Figure 6C). The gene expressions of TRAP, cathepsin K, MMP-9, and RANK also increased in the differentiated osteoclasts with IgG stimulation, however, the stimulatory effects of IgG were also smaller than CRP (data not shown).

## Discussion

In RA, high CRP levels correlate to rapid and severe progression of joint damage in one year, and persistently high CRP levels are associated with substantial progression in radiological joint damage [12]. Because CRP plays a pathogenic role in the inflammation observed in RA, it is possible that CRP is associated with the bony destructive process in the pathogenesis of RA. However, the pathogenic mechanisms of CRP in bone destruction have not been investigated. In this study, we studied the role of CRP on bone destruction of RA, especially its effect on RANKL production and osteoclast differentiation.

To determine CRP expression in the synovial tissues, we measured CRP levels in the SF and synovium of RA patients. SF CRP levels were higher in RA patients than in OA patients. There was a strong correlation between serum and SF CRP levels in RA patients. The serum CRP level was similar and correlated to the SF CRP level, suggesting that elevated SF CRP is mainly caused by a systemic response rather than a local response at the joint level, because many inflammatory molecules show higher SF levels than serum levels. SF monocytes and lymphocytes are accessorial sources of SF CRP; however, the main source of SF CRP is an inflow of serum CRP, which is produced by hepatocytes. Thus, serum CRP level proves to be a useful marker in RA because it reflects both systemic and local inflammatory responses.

To determine the direct pathogenic effect of CRP on bone destruction in RA, we studied the effect of CRP on the production of RANKL from monocytes, which is a key molecule in osteoclastogenesis in RA. When CD14+ monocytes were isolated from peripheral blood and stimulated with CRP, the expression and production of RANKL increased. CRP did not increase the production of IL-1β, IL-6, and TNF-α in monocyte culture, indicating that CRP directly stimulates monocytes to produce RANKL. This phenomenon is not mediated by the proinflammatory cytokines, which are known to stimulate CRP production in hepatocytes and induce RANKL expression in RA.

To determine the direct effect of CRP on osteoclast differentiation, peripheral blood monocytes were cultured with CRP and M-CSF in the absence of RANKL. In RA, bone destruction is mainly regulated by osteoclasts, and RANKL is an essential molecule for the induction of osteoclastogenesis in peripheral blood monocytes [27]. However, we found that CRP, in combination with M-CSF, induced osteoclast differentiation in the absence of RANKL, an observation suggesting that CRP could substitute for RANKL in the induction of osteoclastogenesis. We suppose that CRP is induced by IL-6, IL-1, and TNF-α in the inflammatory condition of RA, and CRP independently stimulates RANKL expression and osteoclast differentiation from osteoclast precursors in synovial tissues and synovial fluid. It is possible that CRP could potentiate the osteoclastic effect of the proinflammatory cytokines, and further investigation is required to evaluate the connection to RA synovial tissues.

Finally, we investigated the pathways of CRP-induced RANKL expression and osteoclastogenesis. On monocytes and macrophages, CRP is mostly mediated by FcγR- dependent pathways. High-affinity receptor FcγRI (CD64) and low-affinity receptor FcγRIIA (CD32) are activating receptors, while low-affinity receptor FcγRIIB (CD16) is an inhibitory receptor [28,29]. CRP activates FcγRs, which phosphorylate immunoreceptor tyrosine-based activating motifs, causing the activation of Syk (spleen tyrosine kinase) and in turn initiating downstream signaling cascades [29]. The interaction of CRP and FcγRs promotes survival and proliferation of macrophages and enhances the release of proinflammatory molecules through stimulation of MMPs, MCP-1, and M-CSF expression and inhibition of IL-10 secretion [30-34]. In RA animal models, FcγRs directly mediate cartilage destruction. FcγRIIb^−/−^ and FcγRI/II/III^−/−^ mice enhance bone erosion and osteoclast numbers as well as severe joint inflammation in antigen-induced arthritis, suggesting that the net effect of FcγRs on bony destruction could be primarily mediated by downregulated FcγRIIb [35-37]. In this study, the blockage of all three FcγRs partially abrogated the CRP-induced RANKL expression and osteoclast differentiation through the inhibition of osteoclast fusion proteins such as DC-STAMP and MFR, suggesting all three receptors were involved in the osteoclastogenic effect of CRP. The process of bone destruction in RA is very intricate, involving complex interactions between various cytokines and cells. The relationship between CRP and FcγRs requires further exploration in terms of the network effects and the mechanisms of CRP and FcγRs in osteoclastogenesis in RA.

## Conclusions

CRP induced RANKL expression and stimulated osteoclastogenesis and bone resorbing function from osteoclast precursors. In treating patients with RA, the significance of reduced serum CRP levels lies not only in controlling disease activity, but also in providing the possibility for prevention of bony destruction.

## Abbreviations

BSA: bovine serum albumin
CRP: C-reactive protein
CTR: calcitonin receptor
DC-STAMP: dendritic cell-specific transmembrane protein
ELISA: enzyme-linked immunosorbent assay
ESR: erythrocyte sedimentation rate
FBS: fetal bovine serum
FcγR: Fcgamma receptors
ICAM: intercellular adhesion molecule
IgG: immunoglobulin G
IL: interleukin
MCP: monocyte chemotactic protein
M-CSF: macrophage colony-stimulating factor
MEM-α: minimal essential medium alpha
MMP: matrix metalloproteinases
OA: osteoarthritis
PBMC: peripheral blood mononuclear cell
PBS: phosphate-buffered saline
RA: rheumatoid arthritis
RANKL: receptor activator of nuclear factor kappa-B ligand
rh: recombinant human
SF: synovial fluid
TTBS: Tween 20 in Tris-buffered saline
TNF-α: tumor necrosis factor alpha
TRAP: tartrate-resistant acid phosphatase
VCAM: vascular cell adhesion molecule
